# Enhanced Raman Scattering in CVD-Grown MoS_2_/Ag Nanoparticle Hybrids

**DOI:** 10.3390/ma17174396

**Published:** 2024-09-06

**Authors:** Dionysios M. Maratos, Antonios Michail, Alkeos Stamatelatos, Spyridon Grammatikopoulos, Dimitris Anestopoulos, Vassilis Tangoulis, Konstantinos Papagelis, John Parthenios, Panagiotis Poulopoulos

**Affiliations:** 1 Department of Chemistry, University of Patras, 26504 Patras, Greece; up1070376@upatras.gr (D.M.M.); vtango@upatras.gr (V.T.); 2 Department of Physics, University of Patras, 26504 Patras, Greece; michailantonios@gmail.com; 3 Institute of Chemical Engineering Sciences, Foundation for Research and Technology-Hellas, 26504 Patras, Greece; anestopd@gmail.com (D.A.); kpapag@physics.auth.gr (K.P.); 4 Department of Materials Science, University of Patras, 26504 Patras, Greecespiridongramma@upatras.gr (S.G.); 5 Department of Mechanical Engineering, University of Peloponnese, M. Alexandrou 1, 26334 Patras, Greece; 6 School of Physics, Department of Solid State Physics, Aristotle University of Thessaloniki, 54124 Thessaloniki, Greece

**Keywords:** SERS, LSPRs, MoS_2_, Ag nanoparticles

## Abstract

Surface-Enhanced Raman Spectroscopy (SERS) is a powerful, non-destructive technique for enhancing molecular spectra, first discovered in 1974. This study investigates the enhancement of Raman signals from single- and few-layer molybdenum disulfide (MoS_2_) when interacting with silver nanoparticles. We synthesized a MoS_2_ membrane primarily consisting of monolayers and bilayers through a wet chemical vapor deposition method using metal salts. The silver nanoparticles were either directly grown on the MoS_2_ membrane or placed beneath it. Raman measurements revealed a significant increase in signal intensity from the MoS_2_ membrane on the silver nanoparticles, attributed to localized surface plasmon resonances that facilitate SERS. Our results indicate that dichalcogenide/plasmonic systems have promising applications in the semiconductor industry.

## 1. Introduction

Raman spectroscopy serves as an effective, non-destructive technique for material characterization by probing molecular vibrations and phonon modes within crystalline structures. However, in field testing conditions, the method’s analytical capabilities are diminished due to its low sensitivity, which arises from signal attenuation and the inherently small extinction cross-sections of the analytes. The phenomenon of Surface-Enhanced Raman Spectroscopy (SERS) shows great potential as a technique to enhance signal quality and sensitivity, making it more easily applicable and potentially capable of achieving even single-molecule detection [1]. The mechanisms responsible for signal enhancement in SERS involve both chemical and electromagnetic effects, which are often challenging to distinguish from one another. The electromagnetic mechanism, which is the dominant and most well-understood in SERS, occurs when a laser beam irradiates a metallic surface with free electrons. This laser frequency resonates with the collective oscillation frequency of the conduction band electrons, enhancing the Raman signal [2]. Also, in specific areas known as “hot spots”, a strong local field amplification is produced around the metallic interface due to the light entrapment, which causes an oscillating dipole to be formed around the noble metal nanoparticles (NPs), thus enhancing the intensity of the outcoming radiation [3]. The interface chemistry between the NPs and MoS_2_ may also play an important role in the modification of the contact resistance, which is crucial for applications [4,5]. However, this subject goes beyond the scope of the present manuscript.

Given the affinity between substrate and analyte, electromagnetic enhancement is a tunable parameter for refining the SERS method by controlling the thin films’ NP growth parameters. However, the self-assembly process used to grow the nanoparticles leads to an inhomogeneous distribution on the substrate. The lack of uniform production methods of thin films leads to randomly placed hot spots, while a partial oxidation of silver plasmonic NPs may further affect the stability of the system.

The chemical mechanism, which is less well understood, is observed primarily on the surfaces of adsorbed molecules. It primarily involves charge transfer mechanisms where the wavelength of the excitation resonates with the metal–ligand complex charge transfer electronic states. Charge transfer between the metal surface and the absorbed molecule is considered to be a resonant effect that can enhance the Raman signal by roughly 10^4^. This enhancement is dependent on the application of an electrical potential. When the energy gap between the Fermi and the electronic level of the noble metal matches the energy of the laser, photon/electron transfer occurs, leading to a pronounced Raman signal [6]. The enhancement depends on the nature of the material, composition, geometry, and aggregation state, as these factors directly influence plasmon oscillation. Noble metal nanoparticles are known to exhibit localized surface plasmon resonances (LSPRs) in the visible spectrum, making them widely used in SERS applications [7]. Other factors affecting the phenomenon include the manufacturing conditions, the orientation of the adsorbate, and the surface roughness.

While a major limitation of SERS in quantitative analysis is its poor reproducibility, recent advancements in the development of relatively homogeneous substrates have addressed this issue. Another challenge during SERS is the potential interference from fluorescence, which can obscure the specific spectra of the materials being analyzed. It has been demonstrated that MoS_2_, a two-dimensional chalcogenide material, can generate SERS phenomena when in nanosheet form [8]. MoS_2_ is increasingly valued for its ideal properties in optoelectronic and biosensing applications [9], and is suitable for stabilizing metal nanoparticles to form composites. Noble metal NPs also possess unique properties that make them optimal for these applications, suggesting that combining MoS_2_ with noble metals could enhance their capabilities. We have previously shown that that highly crystalline single- and few-layer MoS_2_ can be synthesized via chemical vapor deposition using sodium molybdate (Na_2_MoO_4_) as a wet precursor, employing a novel, controllable, and eco-friendly method [10]. The produced MoS_2_ nanosheets were transferred on a silver thin film, which were prepared via physical vapor deposition and post-annealed to form nanoparticles. The MoS_2_ on Ag NP nanocomposites exhibited enhanced SERS features due to an increased quantity of the hot spots. As mentioned earlier, in the electromagnetic mechanism of SERS, the laser frequency matches the oscillation frequency of conduction band electrons, enhancing the Raman signal. In “hot spots”, where strong local field amplification occurs around the metallic interface, the signal is further boosted. Our goal is to demonstrate that SERS in monolayer and bilayer MoS_2_ is activated at these hot spots, which are characterized by higher strain and charge accumulation. To achieve this, we elaborate a Raman-spectroscopy-based method to measure strain and doping in MoS_2_ on Ag NP nanocomposites.

The Raman spectrum of multilayer MoS_2_ features two prominent Raman peaks at 383 cm^−1^ and 408 cm^−1^, corresponding to the E_2g_ and A_1g_ vibrational modes, respectively [11]. The E_2g_ mode is a doubly degenerate in-plane vibration, and the vibrations of sulfur (S) and molybdenum (Mo) atoms are in anti-phase, whereas the A_1g_ mode is a non-degenerate out-of-plane vibration involving only vertical vibrations of the sulfur atoms relative to the layer [12]. As the number of layers decreases, the A_1g_ mode softens due to weakened interlayer coupling, whereas the E_2g_ mode stiffens because of enhanced dielectric screening [12,13]. It is a common practice to identify the number of layers by measuring the frequency difference Δω between the A_1g_ and E_2g_ modes [13,14]. The reduction in symmetry changes the phonon assignment from E’ and A’ in even-layered MoS_2_, to E_2g_ and A’ in odd-layered MoS_2_ such as monolayer MoS_2_ [12,15]. To simplify notation, we will use the monolayer assignments E’ and A’ regardless of the number of layers.

## 2. Materials and Methods

Synthesis of the 2D MoS_2_ flakes was carried out through chemical vapor deposition (CVD), by the reaction of sodium molybdate (Sigma-Aldrich, Darmstadt, Germany. 331058) with sulfur (Sigma Aldrich n.84683). More specifically, a sodium molybdate aqueous solution was spin-coated for 120 s at 2000 rotations per minute (rpm) onto quartz and Si/SiO_2_ wafers, which were previously cleaned with organic solvents and treated using an oxygen plasma procedure. The coated wafer was then placed at the center of a quartz tube positioned in a high-temperature furnace with a diameter of 1 inch. The crucible containing the sulfur powder was placed at the edge of the quartz tube in an independent zone heated by a heating mantle, outside the furnace, from the side where the purging nitrogen gas flows at a rate of 50 sccm. The temperature of the furnace zone was then increased with a steady ramp of 13 °C/min until it stabilized at 800 °C. Afterwards, the independent zone was heated to 250 °C in order for the sulfur to melt, with a rate of 60 °C, and then stabilized for 15 min. The sulfur vapors were carried through the quartz tube by the nitrogen gas flow and ended up at the fume hood. After 15 min, the furnace was shut down and the quartz tube was immediately removed for cooling at room temperature. The whole procedure was carried out with the quartz tube shut at both edges with flanges that allowed nitrogen to flow in one direction under atmospheric pressure.

Silver thin films with nominal thickness *t* varying between 10 and 15 nm were deposited on quartz glass substrates at room temperature (RT) or at 420 °C using direct current (DC) magnetron sputtering. The base pressure of the medium vacuum sputter coater device (custom-modified Balzers Union model SCD040, Oerlikon Balzers, Balzers, Liechtenstein) was 1.5 × 10^−2^ mbar. During deposition, the pressure increased to 5 × 10^−2^ mbar due to the insertion of sputtering gas (argon) through a fine valve. The deposition rate was kept at about 0.35 nm/s. The fabrication of the silver NPs was achieved by annealing the thin films, which were deposited at RT in a furnace with air at 460 °C. A muffle furnace was used for the annealing (model Linn elektronik VMK 22, Escenfelden, Germany). For deposition at 420 °C, silver NPs were formed directly during the deposition time due to the elevated temperature of the sample holder. Response to optical and ultraviolet light was recorded at RT by means of a Perkin-Elmer λ-35 UV-Vis spectrometer (PerkinElmer, Akron, OH, USA) working at the wavelength range of 200–1100 nm. The UV-Vis spectra were recorded in transmission geometry.

The experimental procedure involved the fabrication of three different samples in total.

Sample 1 (MoS_2_ on Ag NPs): Initially, silver thin films with *t* = 10 nm were deposited for 30 sec on quartz substrate at RT and subsequently annealed to produce NPs. MoS_2_ 2D membrane was produced by CVD on a 300 nm SiO_2_ and transferred onto the silver NPs lying on the quartz substrate NPs by means of the wet transfer method. In particular, a 200 nm PMMA thin film (Micro Chem PMMA 495K A3, NEWTON, MA, USA) was spin-coated on the as-grown MoS_2_ and the stack was then immersed into triply deionized water (ρ = 18.2 ΜΩ cm), obtained through a tabletop ultrapure water production system (Synergy UV, Merck KGaA, Darmstadt, Germany). The edges of the film were lightly poked with tweezers to allow water to penetrate between the PMMA and SiO_2_. Due to the hydrophilic nature of SiO_2_, the MoS_2_ film was separated from the SiO_2_ substrate but remained attached to the PMMA film. The PMMA/MoS_2_ stack was then placed onto the Ag nanoparticle-coated quartz substrate. Finally, the PMMA film was removed by exposing the sample to hot acetone vapors [16].

Sample 2 (Ag NPs on MoS_2_): This sample consisted of an initial layer of MoS_2_, which was produced by CVD, transferred onto a quartz substrate followed by the deposition of silver via sputtering. The MoS_2_/quartz substrate was kept at 420 °C during the silver deposition for 30 sec. This process is adequate for silver NPs to be formed on substrates such as glass or quartz or MoS_2_ because the surface energy of those substrates is much smaller than that of Ag [17,18].

Sample 3 (MoS_2_ on annealed Ag NPs): Ag NPs were formed on a quartz substrate by sputtering with the substrate temperature at 420 °C for 45 sec. MoS_2_ crystals were again grown by CVD separately on a Si/SiO_2_ wafer and then transferred via the PDMS stamping method [19]. More specifically, a PDMS stamp was attached to the Si/SiO_2_ wafer containing the as-grown MoS_2_. The stack was again immersed in water and the stamp was slowly released from the substrate. Similar to the PMMA-based transfer used in sample 1, the triply deionized water interpenetrated the MoS membrane/SiO_2_ interface, resulting in the detachment of the MoS_2_ membrane from the SiO_2_ substrate. Finally, the PDMS/MoS_2_ stack was gently stamped onto the quartz substrate covered with the Ag NPs. PDMS was then slowly removed, releasing the MoS_2_ membrane onto the Ag NPs.

Raman spectra were collected in the backscattering geometry using a Renishaw InVia 2000 spectrometer equipped with a 2400 groove per mm grating, providing a spectral resolution of nearly 2.5 cm^−1^ with spectral accuracy better than 0.1 cm^−1^. In order to avoid unintentional heating of the sample, the excitation power was kept below 200 µW in all measurements. The Raman spectra were fitted with Lorentzian peaks using the software WiRe 4.2 by Renishaw, Gloucestershire, UK.

Surface topography images of the sample were acquired by the FSM Nanoview 2000 (Suzhou FlyingMan Precision Instruments Co., Ltd., Suzhou, China) operating in tapping mode. The cantilever used was beam-shaped silicon supplied by Budget sensors with a nominal force constant of 48 N/m and a tip radius of ~10 nm. Image processing was carried out using open-source software: WSxM version 3.1 (Nanotec Electronica, Madrid, Spain) and Gwyddion version 2.46 (CMI, Brno, Czech Republic).

## 3. Results

### 3.1. First Sample: MoS_2_ Wet-Transferred on Ag NPs Sputtered and Annealed on Quartz

**Figure 1:**
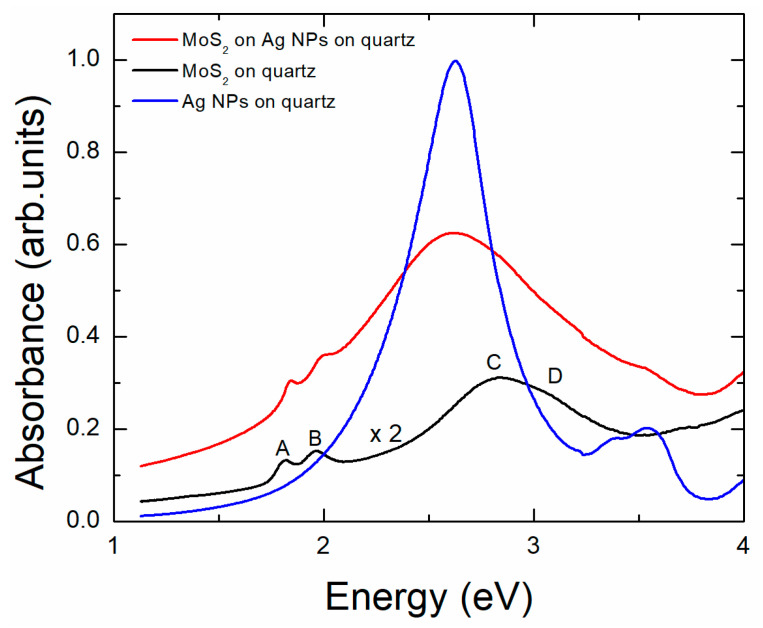
UV-Vis absorption spectra of the various samples on quartz substrate. The absorption spectrum of MoS_2_ on quartz substrate (black line) has been multiplied by 2 for better visibility.

In Figure 1, we show the optical density (absorbance) of MoS_2_ on quartz. The selection of quartz as a substrate allows for spectral recording at least up to 5 eV, as quartz is practically transparent across the entire range. To ensure precise results, we first recorded the transparency T of the films and then normalized this by dividing it by the transparency T_S_ of the substrate [20]. Finally, we used the formula absorbance = −log(T/T_S_). The absorbance spectrum of Figure 1 shows a doublet of maxima A and B. This doublet is due to the excitonic transitions in K point of the Brillouin zone of few-layered MoS_2_ [21,22]. On the other hand, the broader maximum at about 2.8 eV, consisting of the doublet C and D, is due to excitonic transitions in regions with a high density of states within Brillouin zone, e.g., P and Q points [23]. The direct band gap of MoS_2_ is about 2.1 eV [24]. In Figure 1, we also show the absorbance of MoS_2_ on quartz covered by silver nanoparticles (NPs). For direct comparison, we include the absorbance spectra of Ag NPs on quartz before the deposition of MoS_2_. The Ag film before annealing had a thickness of 10 nm. After annealing, self-organized Ag NPs were formed on the quartz surface to minimize the surface energy of the system [25]. The spectrum of Ag NPs consists of an intense peak at about 2.6 eV and a smaller one, sometimes appearing as a doublet, at about 3.5 eV. The first peak is the main localized surface plasmon resonance (LSPR) of Ag NPs. The second is a higher-order mode. The origin of the second peak is discussed in Ref. [17]. Interestingly, the spectrum of MoS_2_ combined with Ag NPs shows a superposition of the MoS_2_ and Ag NP characteristics. The characteristic doublet peaks A and B of MoS_2_ remain at their original positions. The MoS_2_ peak at 2.8 eV overlaps with the main LSPR peak of Ag NPs, which broadens without a significant shift. In contrast, the higher-order LSPR peak at 3.5 eV of the Ag NPs nearly fades away. This indicates an interaction between MoS_2_ and Ag NPs that affects the overall absorbance of the system. One would generally expect the LSPR of Ag to exhibit a notable red shift due to the increase in the refractive index as air is replaced by MoS_2_ [26]. The absence of this red shift may suggest inadequate coverage of Ag by MoS_2_. This observation will be further explored in the next paragraph when discussing the Raman signal.

**Figure 2:**
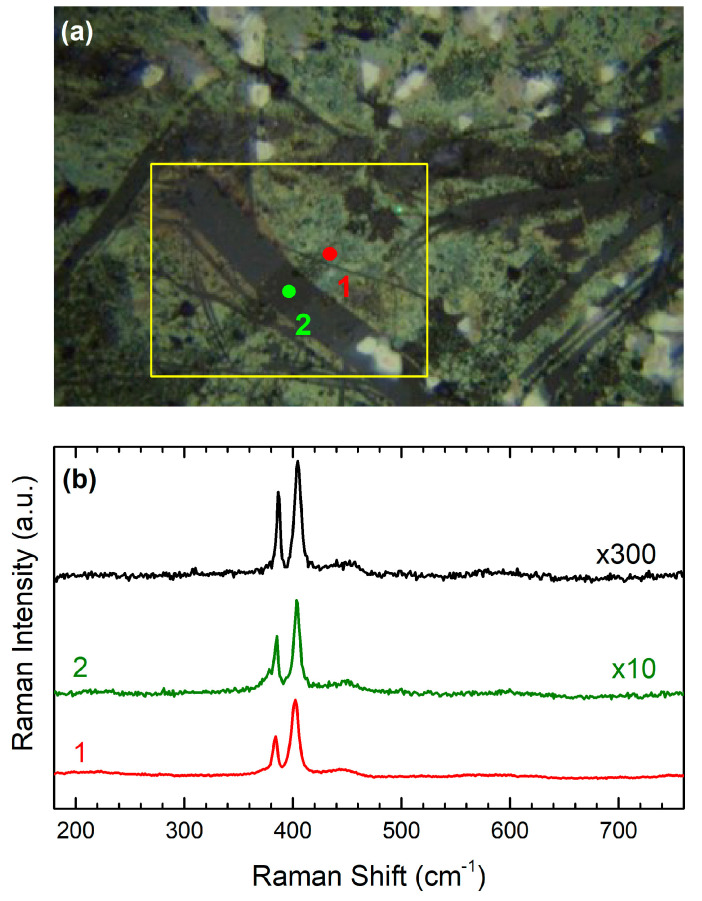
(**a**) Optical microscopy image of sample 1 (inset: schematic of its structure). The yellow line rectangle delineates the Raman mapping window of 80 × 68 μm^2^. Points 1 and 2 lie within the monolayer region as shown in Figure 3b. (**b**) Raman spectra of monolayer MoS_2_ recorded from the spots shown in (**a**) in comparison with monolayer MoS_2_ on quartz (black line).

In Figure 2, we present a characteristic Raman spectrum of monolayer MoS_2_ on bare quartz (black solid line). The spectrum features two prominent peaks, namely, the E’ mode, located around 383 cm^−1^, which corresponds to the in-plane vibrations of sulfur atoms, and the A’, located around 407 cm^−1^, which corresponds to the out-of-plane vibrations of molybdenum atoms. The difference Δω = Pos(A’) − Pos(E‘), where Pos(A’) and Pos (E’) are the peak positions of the A’ and E’ Raman bands, respectively, is commonly used to determine the thickness of the MoS_2_ crystal [13]. As the layer thickness decreases to a single layer crystal, the in-plane mode shifts closer to 386 cm^−1^, while the out-of-plane mode shifts towards 404 cm^−1^ [27]. The observed peak differences in the spectra are consistently less than 20 cm^−1^, confirming the monolayer thickness of the analyzed regions. The total Raman signal, TRS, (TRS = I(E’) + I(A’), where I(E’), I(A’) are the peak heights of E’ and A’ Raman bands, respectively, was found to be 10 times more intense compared to areas with no signal amplification and 300 times more intense compared to the MoS_2_ on bare quartz (Figure 2). Based on the above, we conducted a detailed Raman mapping, recording 1435 spectra at 2.0 μm intervals within the yellow rectangular area shown in Figure 2a.

**Figure 3:**
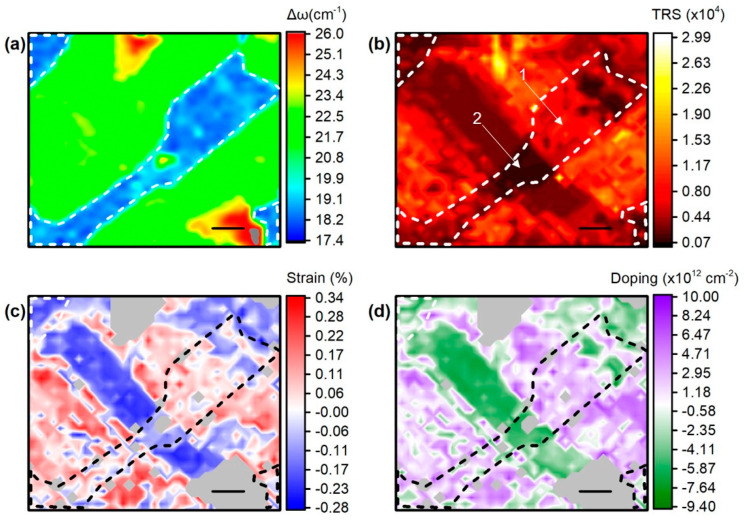
Heat maps of (**a**) peak difference, Δω, (**b**) total Raman signal (TRS) in arbitrary units, (**c**) strain, and (**d**) charge density in sample 1. The dashed area denotes the 1 L area of the mapping window. The numbered arrows in Figure 3b point to the spots shown in Figure 2a.

In Figure 3a, a contour plot of Δω for the Raman mapping region is presented in order to identify the crystal thickness. Blue and teal regions indicate monolayer MoS_2_, with Δω ranging from approximately 17.5 to 20 cm^−1^. The green area, which covers the majority of our mapping window, corresponds to bilayer MoS_2_, with Δω values between 20 and 22 cm^−1^. The yellowish and red areas represent thicker layers of MoS_2_, with Δω values exceeding 22 cm^−1^. By comparing Figure 2a and Figure 3a, points 1, 2, and 3 lie within the monolayer region.

Figure 3b presents the total Raman signal (TRS) of the probed area. As established in previous studies [28], a thicker sample generally yields a higher TRS, so one might expect a contour plot similar to that of Δω (Figure 3a). However, the TRS contour plot in Figure 3b reveals lower signal intensities in regions with bilayer and trilayer MoS_2_, particularly in the distinct diagonal dark area extending from the top left edge to the bottom right. Additionally, variations in TRS are also observable within the monolayer regions spanning from the top left edge to the bottom right in Figure 3b. By comparing Figure 2a and Figure 3b, it can be observed that point 1 is located in a region where monolayer MoS_2_ is attached directly to the substrate without any Ag nanoparticles beneath it. Point 2, although situated on the monolayer with Ag nanoparticles, exhibits a low TRS signal because it is positioned within a dark TRS spot in an otherwise high TRS area (Figure 3b). Finally, point 3 is positioned on the monolayer with Ag nanoparticles and corresponds to a region with a high TRS signal, identified as a SERS hot spot.

We attributed signal amplification (high TRS) in monolayer to the SERS effect. This effect arises from the concentration of electrons due to plasmonic phenomena occurring when the laser beam interacts with the silver nanoparticles, leading to localized amplification of the Raman signal. Using the methodology from our previous work [29], we analyzed the collected Raman data to determine the strain and doping states of single-layer MoS_2_ as influenced by the interaction with the nanoparticles in the mapped region. The results are presented in Figure 3c,d, which display the strain and doping values, respectively.

**Figure 4:**
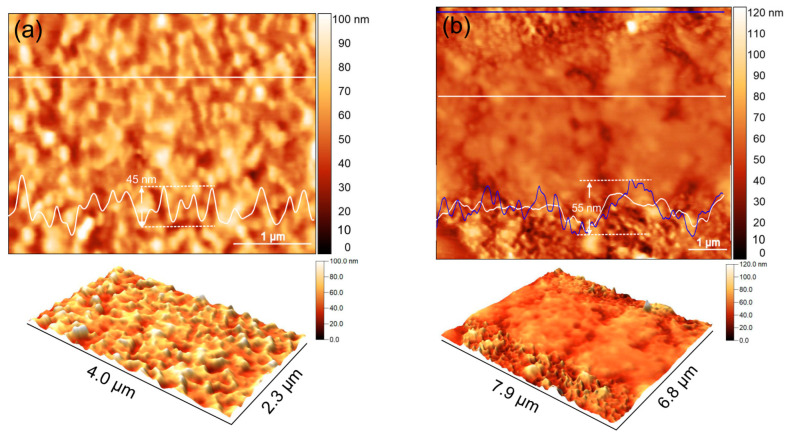
AFM topography images of monolayer MoS2 onto Ag nanoparticles. (**a**—**upper** panel) Wrinkling of the MoS2 membrane induced by Ag nanoparticles. (**a**—**lower** panel) The 3D image of the percolating network of wrinkles corresponding to (**a**). (**b**—**upper** panel) Delamination with increased membrane thickness or incomplete conformation occurs in the mid region. (**b**—**lower** panel) The 3D image of (**b**—**upper** panel) clearly showing the delaminated area. Characteristic line profiles are also included in the topography images.

By correlating the areas with TRS amplification (Figure 3b) with the strain (Figure 3c) and doping (Figure 3d) maps, we deduced that the amplified TRS on the monolayer is located on areas with higher charge density as well as strain. Similar observations were made for the bilayer areas, which cover most of the mapping window (green areas in Figure 3a). It is interesting to note that higher strain is expected in regions with higher mechanical interaction between the Ag nanoparticles and the overlaying MoS_2_ sheet, as will be demonstrated in the AFM topography images (Figure 4).

Figure 4a,b depict the formation of wrinkles in monolayer MoS2 induced by the presence of Ag nanoparticles, as visualized through AFM topography images. Similar to graphene, the wrinkling morphology of the MoS2 monolayer is determined by the density and size of the Ag nanoparticles [30]. In areas with low nanoparticle density, the monolayer MoS2 adheres closely to the substrate, with detachment only occurring in small regions around the nanoparticles. As the nanoparticle density increases, wrinkles begin to form, linking adjacent nanoparticles. In the analyzed samples (1 and 3), the nanoparticle density results in a percolating network of wrinkles across the sample (Figure 4a). The conformation of the monolayer on the nanoparticles induces strain and excess charges in the MoS2 membrane [31]. However, when the MoS_2_ membrane thickness is increased, or if the membrane does not fully conform to the nanoparticles due to the transfer process, global delamination from the substrate is observed (Figure 4b). This delamination leads to strain relaxation and a decrease in charge density within the membrane. The roughness of the line profiles in Figure 4a,b (upper panel) can indicate how well the membrane conforms to or delaminates from the nanoparticles. Specifically, the roughness in well-conformed areas is greater than 6 nm, while in delaminated areas, it is less than 1.5 nm. This difference is clearly shown by comparing the profile lines in the upper panel of Figure 4b.

### 3.2. Sample 2: Ag NPs Grown in Situ on Top of CVD-Produced MoS_2_ Membrane Transferred on Quartz

**Figure 5:**
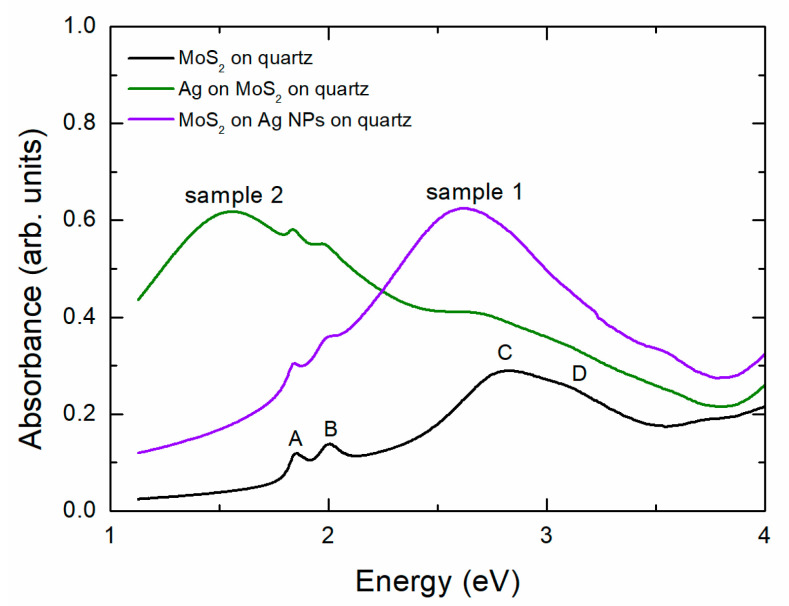
UV-Vis absorption spectra of various samples, as indicated, on quartz substrate.

In Figure 5, we show the absorbance spectra for the second sample. The spectrum of MoS_2_ on quartz for this sample is similar to that of sample 1. Notably, the spectrum of MoS_2_ on quartz covered by Ag nanoparticles (NPs) reveals significant features. Given that MoS_2_ has a lower surface energy than quartz or glass, one would expect Ag NPs to self-organize directly on the heated substrate. The thermodynamic process governing this self-organization is detailed in Ref. [32]. The characteristic features A, B, C, and D of the MoS_2_ spectrum are preserved, indicating that the short in situ annealing process does not cause extensive damage to the MoS_2_ structure. It is important to note that the optical beam covers more than 1 mm^2^ of the sample surface, resulting in a signal that is an average over this area. The main LSPR of Ag shows a considerable red shift. For comparison, Figure 5 includes the spectrum of sample 1, which only exhibits a broadening of the LSPR due to increased inhomogeneity, without any red shift relative to the LSPR of Ag NPs on quartz shown in Figure 1. The fact that the LSPR of sample 2 shows a prominent red shift, which is depicted in Figure 5, indicates a full adhesion of MoS_2_ with the Ag NPs. The higher refractive index of MoS_2_ compared to SiO_2_ contributes to this red shift. We will revisit this observation in the next paragraph when discussing the Raman signal. As we will detail, the Raman signal indicates that single-layer MoS_2_ areas are degraded during the growth process, while thicker layers remain intact, which is why the main features of MoS_2_ in Figure 5 are preserved.

**Figure 6:**
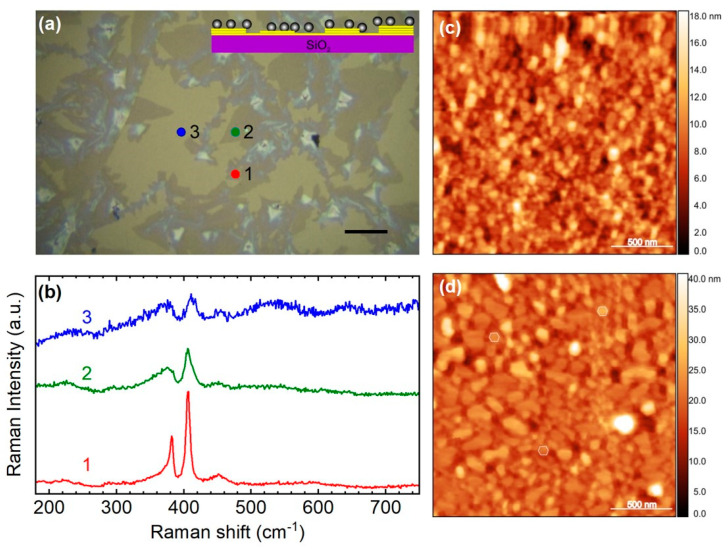
(**a**) Optical microscopy image of sample 2 (inset: schematic of its structure). (**b**) Representative Raman spectra from (1) multilayer, (2) bilayer, and (3) region where the single-layer MoS_2_ was destroyed by heat during the annealing process. (**c**,**d**) AFM images of high-density Ag nanoparticles on MoS_2_ sheet. Ag nanoparticles on MoS_2_, of small lateral dimensions on thinner membrane regions like point 1 (**c**) and larger sizes, occasionally hexagonal shapes on thicker regions like point 2 (**d**).

In Figure 6 spectra from a multi-layered (N ≥ 3), bilayer and monolayer region are compared. The regions are marked as points 1, 2, and 3 in Figure 6a, respectively, and the corresponding spectra are presented in Figure 6b. Trilayer and multilayer areas (Δω ≥ 25 cm^−1^) are present with a strong signal (point 1). Bilayer areas (20 < Δω ≤ 23 cm^−1^) are significantly degraded (point 2), while monolayer regions (Δω ≤ 20 cm^−1^) seem to be totally extinct (point 3). Unfortunately, a TRS comparison for SERS location could not be carried out in this case due to the absence of single-layer areas.

However, it is interesting to assess the grown nanoparticles on locations with varying thicknesses of MoS_2_ membrane. Figure 6c,d show AFM images of Ag nanoparticles grown on the MoS_2_ membrane in sample 2. The nanoparticles exhibit high density and display noticeable variations in shape and size. In Figure 6c, the flat nanoparticles, with random shapes and small lateral dimensions, are observed on thinner regions of the MoS_2_ membrane located on point 1 (Figure 6a). In contrast, Figure 6d shows nanoparticles grown on thicker regions of the membrane located on point 2 (Figure 6a), where most of them are faceted, with some adopting a hexagonal shape. These observations closely align with the findings of Deng Y. et al. [33].

### 3.3. Sample 3: CVD-Grown MoS_2_ Membrane Transferred onto Ag NPs Formed in Situ on Quartz

**Figure 7:**
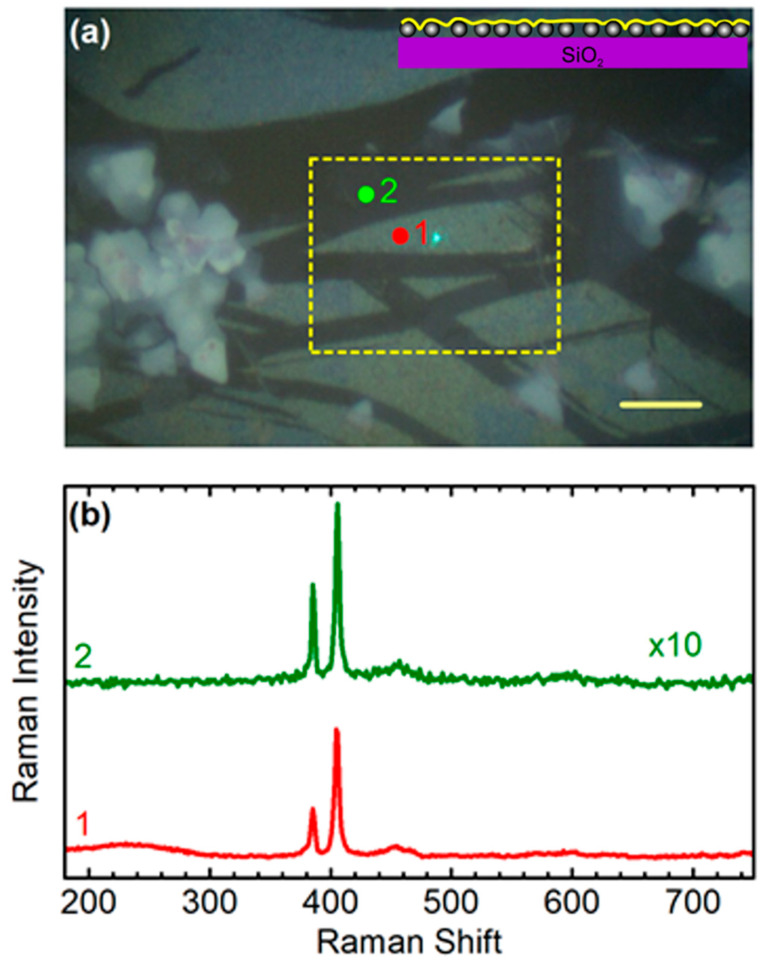
(**a**) Optical microscopy image of sample 3 (inset is a schematic showing its structure). The yellow dashed line rectangle delineates the Raman mapping window of 31.5 × 25.5 μm^2^. (**b**) Representative Raman spectra of MoS_2_ from locations on sample 3 shown in (**a**). 1 (Red) corresponds to areas with underlying NPs and 2 (green) to areas without NPs.

The rectangular area in Figure 7a is the region of interest. The white aggregates on the left and right are multilayer and bulky crystals [10]. Due to low optical contrast on quartz, MoS_2_ monolayers and few layers are not visible. However, the Δω map in Figure 7 clearly identifies monolayer and bilayer regions along with some thicker islands. The signal on the areas covered with NPs is significantly enhanced with a median increase of 10x, as shown in Figure 7. This enhancement might be attributed to the in situ annealing process conducted in a vacuum chamber, which likely resulted in better uniformity of the nanoparticles, or it could be due to the MoS_2_ transfer method using PDMS instead of PMMA, as described in the Section 2.

**Figure 8:**
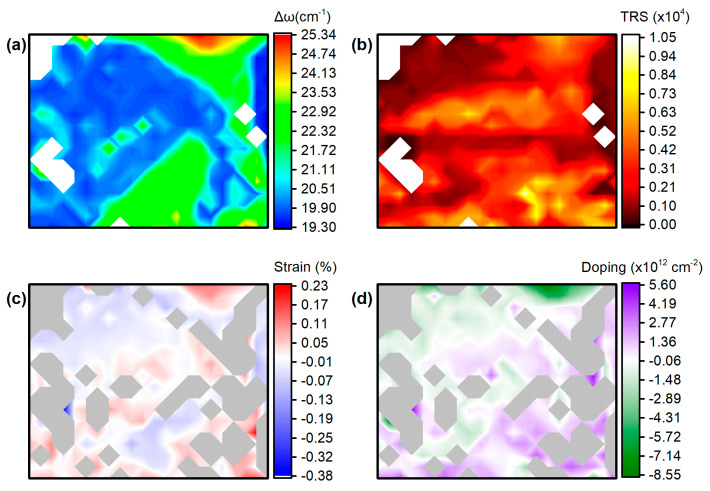
Contour plots of (**a**) peak difference, Δω, (**b**) total Raman signal, TRS in arbitrary units, (**c**) strain and (**d**) charge density in sample 3.

Similar to sample 1, we conducted a detailed Raman mapping, capturing 396 spectra at 1.5 μm intervals within the yellow dashed rectangular area shown in Figure 7a. Figure 8a presents the contour map of the Δω intensity across the mapped region, illustrating the crystal thickness. The teal regions correspond to monolayers with Δω values ranging from about 18.5 to 20 cm^−1^, while the green regions, which dominate the mapping window, represent bilayer areas with Δω values ranging from about 20 to 22 cm^−1^. The TRS contour map in Figure 8b reveals a clear enhancement of the Raman signal in areas where the substrate is covered with Ag nanoparticles. The teal areas in Figure 8a, with a Δω of ~19.5 cm^−1^, exhibit strong TRS, suggesting the presence of an SERS phenomenon. The data in Figs. 8c and 8d indicate the presence of strain and doping features in the area of interest, further supporting the idea of interactions with a conformally coated MoS_2_ sheet.

## 4. Conclusions

Thin MoS_2_ flakes studied using Raman spectroscopy show an increase in Raman signal intensity as the crystal thickness increases. However, MoS_2_ placed on top of Ag nanoparticles show a significantly enhanced signal. In certain areas, referred to as “hot spots”, the Raman signal can be nearly an order of magnitude larger. This enhancement is attributed to the localized surface plasmon resonances exhibited by Ag nanoparticles in the visible spectrum, which interact with the laser radiation and the overlaying MoS_2_ sheet. The greatest enhancement occurs in areas where the MoS_2_ sheet covers larger nanoparticles, resulting in direct contact and notable strain due to the geometrical placement and transfer process. Thus, this work demonstrates the SERS effect facilitated by the LSPRs of noble metal nanoparticles. Our findings show that dichalcogenide/plasmonic systems hold potential for applications in the semiconductor industry.

## Data Availability

Data are contained within the article.
